# Notes

**Published:** 1899-05

**Authors:** 


### NOTES.

The average rise in price of horses this spring is twenty-five dollars per head, and every Eastern veterinarian fully appreciates the fact that not for five years has the demand been so great nor the work of the veterinarian so remunerative.

The experiment in Thuringia, Germany, of feeding horses on a baked bread composed of cooked potatoes and meal will be watched with much interest. It is economical, and the problem of feeding horses on long journeys in war times and in transportation long distances would be greatly simplified if it proves successful.

The range horses of Idaho, Montana, and Washington are destined for slaughter and consumption for food through the reopening of the horse-meat canning establishment at Miles City, Montana.

Minnesota veterinarians have just finished a lively skirmish with woodchuck legislation. Minnesota has one of the best practice acts in America. As this act now stands none but graduates of recognized colleges can come before the Board of Examination. Applicants must not only present a diploma, but must also pass such examination as the board prescribes. The woodchuck bill passed the Senate, was introduced in the House, referred to the Judicial Committee, recommended to pass, and was well up on general orders when it was discovered. The bill was never printed, and few of the Senators who voted for it had any definite idea of what they were passing. This bill was got up by two Senators, each of whom had a man whom he wished to have registered. This could not be accomplished without opening up the entire Practice Act so as to compel the Examining Board to register non-graduates. The bill was discovered quite accidentally, and the Twin City veterinarians made an active personal fight in the Legislature. Members of the State Association were telegraphed, and they in turn wired their Representatives and Senators that the bill was bad and ought to be defeated. The result, secured within a few days after the promoters of this bill felt absolutely certain of success, was a vote of 20 to 46 in the House against the bill.

There is a lesson in this. Every State that has a good Practice Act should have a committee of two or three veterinarians selected by the State Association for the express purpose of watching legislation. The members of this committee should preferably reside in or near the capital city.

Grand Rapids, Mich., after much discussion, created the position of milk-inspector and dairy-inspector in 1898, and immediately proceeded to make nugatory all the advantages to be so gained by the appointment of a politician, an ex-United States Senator, to fill the place.

At the April meeting of the Chicago Veterinary Society, Dr. James G. Fish considered the conditions of poll-evil, glandular diseases of the throat, obliterated jugulars, and crest-fallen changes in their relation to soundness.

Examinations of all army veterinarians in connection with the requirements of the new army regulations will take place some time in June.

The board charged with the preparation of the examination questions for the army veterinarians will consist of Captains Steever and Johnson, of the Third United States Cavalry, and Dr. Victor A. Norgaard, of the Department of Agriculture, Washington, D. C.

Some one should shake up the War Department at Washington in its continual breaking of the spirit and letter of the law in the selection of veterinary surgeons. A recent employé for veterinary duties was a self-styled "homœopathic horse-doctor"—a graduate of one of Humphrey's specific boxes of never-failing remedies. Surely this has gone far enough.

The new Illinois veterinary law gives the Board of Veterinary Examiners a wide range of authority. They may accept or reject at their own discretion. It is the plain duty of the Livestock Commissioners to exercise the greatest care in selecting veterinarians for these positions.

